# Qualitative realist evaluation of an occupational therapy intervention programme (ABLE), addressing ability to perform activities of daily living among persons with chronic conditions

**DOI:** 10.1186/s12913-023-10498-4

**Published:** 2024-01-03

**Authors:** Vita Hagelskjær, Eva Ejlersen Wæhrens, Cecilie von Bülow, Kristina Tomra Nielsen

**Affiliations:** 1 Occupation Centered occupational therapy, The Parker Institute, Bispebjerg and Frederiksberg Hospital, University of Copenhagen, Copenhagen, Denmark; 2 Occupational Science, User Perspectives and Community-based Interventions, Department of Public Health, University of Southern Denmark, Odense, Denmark; 3 Department of Occupational Therapy, VIA University College, Holstebro, Denmark; 4 Department of Occupational Therapy, University College of Northern Denmark, Aalborg, Denmark

**Keywords:** ADL, Community rehabilitation, Complex interventions

## Abstract

**Background:**

Chronic conditions are associated with problems related to performance of activities of daily living (ADL) stressing a need to develop and evaluate intervention programmes addressing such problems. Hence, the ABLE programme was developed, and its feasibility evaluated. Implementing intervention programmes in community-based rehabilitation settings requires understanding of how the programme works in various contexts. Applying a realist evaluation approach, the aim of this study was to identify and evaluate interactions between contexts, mechanisms, and outcomes in the ABLE 2.0, to confirm, refine, or reject aspects of the initial programme theory.

**Methods:**

Realist evaluation using qualitative data collected in the ABLE 2.0 randomised controlled trial (*n* = 78). Based on the ABLE 2.0 initial programme theory, qualitative realist interviews were conducted among receivers (*n* = 8) and deliverers (*n* = 3) of the ABLE 2.0 in a Danish municipality. Transcripts were coded, and context-mechanism-outcome configurations were extracted and grouped into contiguous themes. Results were then held up against the initial programme theory.

**Results:**

Four contiguous themes were identified including a total of *n* = 28 context-mechanism-outcome configurations: building a foundation for the entire intervention; establishing the focus for further intervention; identifying and implementing relevant compensatory solutions; and re-evaluating ADL ability to finalise intervention. Overall, the ABLE 2.0 initial programme theory was confirmed. The evaluation added information on core facilitating mechanisms including active involvement of the client in the problem-solving process, a collaborative working relationship, mutual confidence, and a consultative occupation-based process using compensatory solutions. Several contextual factors were required to activate the desired mechanisms in terms of supportive management, referral procedures encouraging the problem-solving process, delivery in the client’s home, skilled occupational therapists, and clients feeling ready for making changes.

**Conclusions:**

The ABLE 2.0 represents a coherent problem-solving occupational therapy process, applicable across sex, age, and diagnoses with the potential to enhance ADL ability among persons with chronic conditions, when delivered as part of community-based rehabilitation services. Knowledge about the interactions between contextual factors, mechanisms, and outcomes in the ABLE 2.0 is central in case of future implementation of the programme in community-based rehabilitation settings.

**Trial registration:**

The trial was prospectively registered on www.ClinicalTrials.gov (registration date: 05/03/2020; identifier: NCT04295837) prior to data collection that occurred between August 2020 and October 2021.

## Background

Problems related to performance of activities of daily living (ADL) tasks are associated with chronic conditions [1–9]. People with chronic conditions often report decreased quality of ADL task performance, reflected as increased effort/fatigue, increased use of time, safety risk and need for assistance when performing specific tasks [10], such as increase in time spent on dressing or increased effort and/or fatigue when cooking a meal.

Accordingly, chronic conditions have been defined as “conditions that last a year or more and require ongoing medical attention and/or limit activities of daily living” [11]. ADL involves tasks that most people need to perform in their everyday lives. Personal ADL tasks include self-care tasks such as eating, toileting, grooming and dressing, while instrumental ADL tasks include domestic tasks necessary for independent living such as shopping, cooking, cleaning and doing laundry [12, 13]. A recent study revealed that more than 65% of the Danish population, aged 16 or above, live with one or more chronic condition [14]. As the probability of dying from one of these diseases is decreasing [15], an increasing number of persons is living with diseases often causing problems with ADL task performance. Limitations in ADL associated with chronic conditions may result in decreased quality of life, and reduced energy and time for participation and engagement in other types of wanted or needed activities at home and in the community [16–18]. Besides affecting these persons’ everyday lives, this also entails an increasing financial cost related to community-based rehabilitation and caregiver services [19–21]. Hence, the need for developing effective interventions is urgent.

Attempting to change the everyday lives of persons with chronic conditions into the better by enhancing their ability to perform ADL tasks, the ‘A Better Everyday Life’ research programme was established in 2015. The focal point in ‘A Better Everyday Life’ is development of a complex occupational therapy intervention programme, named ABLE. By following the United Kingdom Medical Research Council’s (MRC) guidance [22] on how to develop and evaluate complex interventions, the ABLE intervention programme was developed [23, 24], feasibility evaluated in terms of content and delivery [25, 26] and pilot tested to prepare for evaluation based on a full scale trial [27]. This resulted in the ABLE intervention programme version 2.0 (ABLE 2.0) [28] and justification for proceeding to evaluation of the programme in terms of effectiveness, process and cost-effectiveness evaluation [26, 27] as recommended for complex interventions [22].

The ABLE 2.0 has been described in detail in previous studies [23, 25–28]. In short, the manualised ABLE 2.0 is a home-based, individualised, 8-week occupational therapy intervention programme, applicable across diagnoses, age, and sex. The programme is to be delivered as part of community-based rehabilitation services. In a maximum of eight sessions, the programme addresses ADL task performance problems among persons with chronic conditions by offering standardised ADL evaluation, client-centred goal setting, individualised intervention sessions building on an adaptational approach, and finally, re-evaluation of ADL ability and assessment of goal attainment.

When initiating the evaluation phase [22], the ABLE intervention programme was well described, tested, accompanied by a manual, and continuously revised. However, considering the nature of complex interventions, knowledge on how the intervention worked in different contexts was still preliminary. Looking into the series of MRC publications on how to develop and evaluate complex interventions [22, 29, 30], there has been an increasing focus on underlying theories of the complex interventions investigated and on the importance of integrating different evaluation models, e.g., outcome and process evaluation. Specifically, the most recent MRC framework [30] recognises the need for more contextualised understandings of how an intervention induces change, for instance by developing a programme theory. The realist evaluation approach is increasingly used in health service research [31], being a form of theory-driven evaluation, addressing the question “what works, for whom, in what circumstances, and how?” [32]. Realist approaches assume that nothing works everywhere for everyone and that context affects programme outcomes [32, 33]. In a realist evaluation the question is not only “what works?” but “how or why does this work, for whom, in what circumstances?”, and it provides a way of gaining a deeper insight into the nature of a complex intervention and in the implementation context [31]. The premise is that the intervention does not work by itself. Rather it works by the way the receivers and deliverers respond to the resources offered by the programme [32]. Introducing the term ‘mechanism’, i.e. the underlying changes in the reasoning and behaviour of persons triggered by the particular contexts [32, 34], a programme is considered to work (or not to work) because deliverers and receivers of the intervention programme make particular decisions in response to the resources and opportunities provided by the intervention programme, causing certain outcomes. Contextual factors are defined as material/ social/ organisational/ economic/ technical/ individual characteristics. Outcome is defined as the result of the interaction between a mechanism and its triggering context [31, 32]. Contextual factors at different levels (i.e. infrastructural, institutional, interpersonal, and individual) [35] may enable or prevent mechanisms from being triggered, which is expressed as context-mechanism-outcome configurations (CMOCs) [31]. Programme theory is central to realist evaluation forming the means to providing plausible explanations of why a certain intervention works or does not work in certain circumstances [32]. Hence, the overall purpose of conducting a realist evaluation of ABLE 2.0 was to reach a deeper level of understanding of the functioning of the ABLE 2.0 by investigating in what circumstances, for whom, how and why the intervention programme functions [31]. The results will contribute to future revision of the ABLE programme theory and thereby support eventual future implementation of the ABLE programme in community-based rehabilitation settings.

## Methods

### Aim

The aim of the present study was to identify and evaluate interactions between contexts, mechanisms, and outcomes in the ABLE 2.0, to confirm, refine, or reject aspects of the initial programme theory.

### Design and setting

The study was designed as a theory-driven qualitative realist evaluation [36–38] to investigate how and in which circumstances ABLE 2.0 may improve the ADL ability among people with chronic conditions. It was conducted alongside evaluation of effectiveness (ABLE 2.0 randomised controlled trial (RCT)), process, and cost-effectiveness of ABLE 2.0. Details of the designs and methods applied were provided in the published protocol [39]. The reporting of the present study follows the RAMESES (Realist And Meta-narrative Evidence Syntheses: Evolving Standards) II reporting standards for realist evaluations [31].

The study was conducted from June to August 2021 in a Danish municipality, counting about 90,000 people. It was conducted among clients having received and occupational therapists (OTs) having delivered ABLE 2.0 as a part of the ABLE 2.0 RCT. Delivery of ABLE 2.0 and data collection among clients took place in the homes of the clients, while data collection among OTs took place in a rehabilitation centre in the municipality.

### Participants and recruitment

Clients were recruited as a sub-sample among the last included clients randomised to receive the ABLE 2.0 in the ABLE 2.0 RCT. Hence, they lived with one or more medically diagnosed chronic condition(s); were aged ≥18 years; lived in their own home; experienced ADL task performance problems; were motivated and ready for making changes in performance of ADL tasks, and for participating in an occupational therapy intervention; and communicated independently and relevantly. Further, for composition of the sub-sample (estimated *n* = 8), the following criteria were applied: ≥three males; ≥four clients with an Assessment of Motor and Process Skills (AMPS) [40, 41] ADL motor ability < 1.0 logits, assessed at baseline in the ABLE 2.0 RCT, indicating the need of moderate to maximal assistance to live in the community; variation in number of sessions received; and variation in age. Further, they should demonstrate variation in outcomes (measured at the final session of the intervention and assessed by Goal Attainment Scaling (GAS) [42, 43]). The AMPS and the GAS will be described in further detail in the following paragraph on the ABLE 2.0 intervention programme.

OTs (*n* = 3) were recruited provided they had delivered ABLE 2.0 in the RCT [39, 44], had ≥2 years of experience working with the study target group, were calibrated AMPS raters, and were trained in delivering ABLE 2.0 by attending a three-and-a-half-day course prior to the RCT. The course consisted of introduction to ABLE 2.0 and the underlying theories and models, practicing the use of instruments in the programme, and training delivery of ABLE sessions.

### ABLE 2.0 intervention programme

The manualised ABLE 2.0 is a systematic, client-centred, eight-week intervention programme, applicable across sex, age, and chronic conditions, delivered by an OT in the client’s home as part of community-based rehabilitation. Standardised instruments and theoretical models are incorporated in ABLE 2.0. The overall structure of ABLE 2.0 is informed by the Occupational Therapy Intervention Process Model (OTIPM) [45], prescribing a problem-solving process. The problem-solving process, informed by OTIPM [45], includes evaluating ADL ability based on both self-report and observation; involving the client in setting goals and clarifying reasons for the identified ADL task performance problems, and in finding and implementing solutions; and re-evaluation [45]. The conceptual model, ‘the Transactional Model of Occupation’ (TMO) [45], describes how the client’s occupations (i.e. meaningful and purposeful doings) has three interwoven elements; occupational performance (i.e. observable aspects), occupational experience (i.e. how the doing is experienced), and participation (i.e. occupational engagement). Further, the TMO frames occupation as a response to several situational elements, including environmental, sociocultural, task, and temporal elements [45, 46]. The Person-Environment-Occupation Model (PEO) [47] explains the complex relationship between person, environment, and occupation supporting the analysis of the ADL task performance, the planning of the intervention, and the communication and collaboration with the client. Hence, TMO and PEO support the client-centred reasoning during delivery of the programme. The ADL-Interview (ADL-I) [48–50] is used for evaluating the client’s self-reported ADL ability. ADL-I is a standardised evaluation tool, used by OTs, to describe and measure self-reported ADL ability [48–50], in terms of physical effort and/or fatigue, efficiency, safety, and independence (ADL-I Performance), i.e. quality of ADL task performance. The AMPS [40, 41] is a standardised observation-based evaluation tool used by OTs to measure the client’s observed ADL ability in terms of physical effort and/or fatigue, efficiency, safety and independence i.e. quality of ADL task performance. ADL-I [48–50] and AMPS [40, 41] are generic instruments to be applied across diagnoses. GAS [42, 43] is a tool for defining and monitoring individual goals. The client is actively involved in defining the goals and describing levels of goal attainment.

ABLE 2.0 consists of a maximum of eight sessions. Session 1 includes ADL evaluations, using the ADL-I [48–50] and the AMPS [40, 41]; and a mandatory dialogue between the client and the OT to determine eventual discrepancy in their perspectives on the quality of task performance during the AMPS [45]. Session 2 includes goal setting, using GAS [42, 43], and clarification of reasons for the identified ADL task performance problems, using PEO [47] and/or TMO [45]. Sessions 3–7 consist of individually tailored intervention sessions combining nine potential intervention components [23], organised based on PEO [47], and building on an adaptational approach [23, 45]. An adaptational approach includes collaboration between the client and the OT in finding compensatory solutions to the ADL problems, and engaging the client in consultation and education (i.e. collaborative decision-making and strategies on how the client can learn to use the chosen compensatory solutions) [45]. Compensatory solutions may include e.g., changes in habits, in the physical environments, or modification to task performance [23] aiming to reduce effort and/or increase efficiency, safety, and independence in ADL task performance. The final session includes re-evaluation of the perceived and observed ADL ability (ADL-I and AMPS) [40, 41, 50] and evaluation of goal attainment using GAS [42, 43].

### Realist evaluation procedures

**Fig. 1:**
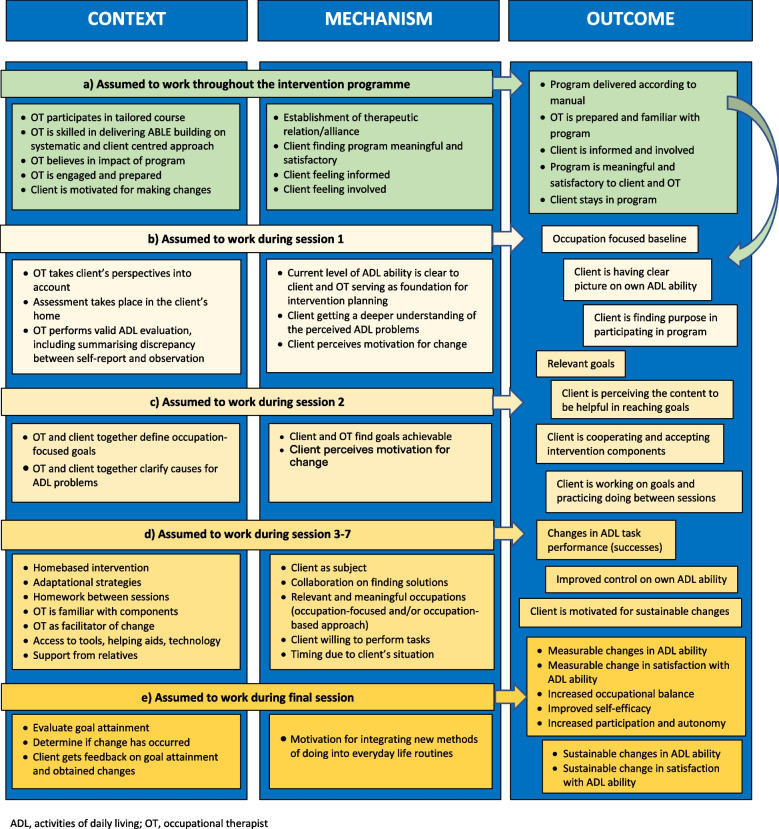
ABLE 2.0 initial programme theory

Following the realistic evaluation cycle [32], the first step was to develop the ABLE 2.0 initial programme theory (IPT), capturing the assumptions of ABLE 2.0 in terms of ‘what works, for whom, in what circumstances, and how?’ [31]. The IPT, illustrated in Fig. 1, was developed based on the theory-of change-logic model [23], constructed during development of the first version of the ABLE intervention programme (ABLE 1.0) [10, 23, 24] and the results of the feasibility study [23, 25, 26]. The overarching IPT was that ABLE 2.0 would improve clinical outcomes in terms of observed and/or self-reported ADL ability, based on a structured and individualised problem-solving process and by applying compensatory solutions in the client’s home.

## Data collection

To evaluate the interactions between contexts, mechanisms, and outcomes, qualitative data based on realist interviews [32, 37] was collected among clients who received and OTs who delivered ABLE 2.0. The interviews were conducted to elucidate aspects of the IPT and to identify emerging CMOCs. Thus, new theory was generated during the process of determining which aspects of the IPT should be confirmed, refined or rejected [51].

### Realist interviews

According to the realist approach, the purpose of an interview is to present the programme theory for the interviewees, for confirmation, refinement or rejection [32, 52]. First, individual interviews were conducted with the OTs, followed by individual interviews with the sub-sample of clients. Finally, a focus group interview with the OTs was conducted. In the individual OT interviews, questions related to their experiences of what (mechanisms), for whom and in which circumstances (context) successes and failures (outcomes) occurred [32]. In interviews with the clients, the questions primarily related to their experiences of whether ABLE 2.0 encouraged them to make changes in reasoning and/or behaviour in relation to ADL task performance (mechanisms) [32]. The final focus group interview with the OTs provided a deeper insight into what was already revealed about the IPT in the individual interviews with OTs and clients [32, 52].

The interviews were conducted in a longitudinal structure, allowing insights from completed interviews to inform the interview guide for the subsequent ones, aiming to further develop and validate the programme theory, as the investigators gained more knowledge [52]. Interview guides were developed and structured to capture in-depth information on programme theory, by using the teacher-learner function [32]. For example, when interviewing the clients on the goal setting process, first the IPT was presented to the client by saying: “A purpose of defining goals in the way it was done, was to encourage you to make a change in your daily life, in order to reduce the problems”. Next, the interviewer asked the client: “Do you think it worked that way? If so, can you say something about what it was that particularly worked for you? If not, will you try to explain why? How did you perceive that the OT listened to your perspective?” Further, the client was asked: “How did you experience the OT’s ability to explain the goal setting procedure to you?” Furthermore, to prompt the participants in remembering details from their sessions, we brought up examples from their intervention e.g., their specific goals, or if the client only received few sessions, a question was: “What happened since you chose to end the programme?” In this way, the realist evaluation approach [32, 52] was reflected in the interview guides as well as during the interviews, to facilitate identification of key contextual differences in outcome patterns [52].

### Data analysis

According to realist evaluation, the analysis took shape as an iterative process [32, 38, 52] and insights were pursued along the way modelling the understanding of the functioning of ABLE 2.0. Data analysis took a ‘retroductive’ approach i.e. “identification of hidden causal forces that lie behind identified patterns or changes in those patterns” [51], using a combination of inductive (i.e., to identify emerging CMOCs in data), and deductive (i.e., to investigate how contextual factors enabled or prevented activation of the desired mechanisms expressed in the IPT) reasoning seeking evidence to confirm, refine, or reject the IPT [51]. Using the standards by Wong et al. [31] and inspired by Gilmore et al. [53], the analysis was carried out as a five step process. First, each interview recording was listened through, and the transcripts were read to gain an overview of the data. Second, each interview was examined and coded in terms of contextual factors, activated mechanisms, and perceived outcomes. Further, paragraphs reflecting CMOCs were extracted. Third, the extracted paragraphs from each type of interview (i.e., client interviews, OT interviews, and focus group interview) were merged, resulting in three matrices. Fourth, to group the data into contiguous units (i.e., themes) across the matrices, and extract theory in terms of CMOCs (i.e., found in more than one data source, expressed with emphasis, or perceived to cause particularly positive or negative changes), two researchers examined the content individually, and then discussed until consensus on themes was reached. Finally, the results were compared to the content of the IPT, to determine which aspects should be confirmed, refined, or rejected and which CMOCs offered the most robust explanations of the observed patterns of outcomes.

Results were presented using the identified themes as a structure. Revealed CMOCs were presented in tables followed by descriptions of how ABLE 2.0 functioned, i.e., interactions between contexts, mechanisms, and outcomes as derived from data, and determination of whether aspects of the IPT (Fig. 1) were confirmed, rejected, or to be refined. To emphasize the impact of how contextual factors were found to enable or prevent mechanisms from being triggered, the presentation of the results was structured within four levels of contextual factors; infrastructural, institutional, interpersonal, and individual levels [35]. Results were documented with quotes as follows: OT interviews, numbered OT1–3; client interviews, numbered C1–8; and the focus group interview, FG. Hence, the tables provide overview of the results while the text provide details and transparency.

## Results

### Participants

A total of eight clients and three OTs were included. Characteristics on the interviewed clients are presented in Table 1. In summary, the clients were three men and five women aged between 69 and 85 years, with a variety of chronic conditions, and seven of them with multi morbidity. Four of the clients had an AMPS ADL motor score < 1.0 logits assessed at baseline, indicating the need of moderate to maximal assistance to live in the community [40, 41]. In total, *n* = 22 (median *n* = 3, range 1–5) goals were defined by the eight clients during their attendance in the ABLE 2.0. In *n* = 20 (90.9%) goals the clients reached the expected, more, or much more than expected level of goal attainment. In *n* = 1 (4.5%) goal the client remained at the baseline level. The three OTs were women, aged 35, 38, and 43 years, with 7, 9 and 11 years of experience, respectively, working as OTs with persons with decreased ADL ability following chronic conditions.

**Table 1:** Characteristics on clients who participated in interviews

Client number	Sex	Age	Civic status	Diagnosis	AMPS ADL motor ability at baseline	Number of sessions received
1	Female	84	Living alone	Medical^c^, orthopaedic/musculoskeletal^a^	0.8	3
2	Male	74	Living with partner	Medical^c^, orthopaedic/musculoskeletal^a^	0.7	4
3	Female	69	Living with partner	Orthopaedic/musculoskeletal^a^	1.1	5
4	Female	74	Living alone	Medical^c^, neurological^b^	0.7	5
5	Female	75	Living alone	Neurological^b^	1.1	4
6	Male	70	Living alone	Medical^c^	0.8	4
7	Male	75	Living alone	Medical^c^, orthopaedic/musculoskeletal^a^	1.3	4
8	Female	85	Living with partner	Medical^d^, orthopaedic/musculoskeletal^a^	1.4	4

*ADL* activities of daily living, AMPS assessment of motor and process skills

^a^ ‘orthopaedic/musculoskeletal’ covers arthritis, chronic/long-term pain, and fracture/replacement

^b^ ‘neurological’ covers stroke (i.e., right−/left-sided stroke, subarachnoid haemorrhage, cerebral aneurism) and non-stroke (i.e., cerebral palsy, traumatic brain injury, multiple sclerosis, parkinsonism)

^c^ ‘medical’ covers cardiovascular disease, respiratory disease, diabetes, cancer, and obesity

### Themes and CMOCs

Across the conducted interviews, CMOCs were identified within four themes: building a foundation for the entire intervention; establishing the focus for further intervention; identifying and implementing relevant compensatory solutions; and re-evaluating ADL ability to finalise intervention. A total of *n* = 28 CMOCs were identified and presented in Tables 2, 3, 4, and 5.

**Table 2:** Context-mechanism-outcome configurations (CMOCs): Building a foundation for the entire intervention

CMOC no	Context	Mechanism	Outcome
	**Infrastructural level**		
1	If ABLE 2.0 is delivered in the context of a supportive collaboration between the departments in the municipality in terms of the municipal guidelines for referring to rehabilitation services (i.e., the client’s pathway to intervention), allowing the logic order of the intervention (i.e., assessment prior to goal setting and dialogue on causes) …	… it may activate the client being actively involved in the entire problem-solving process …	… contributing to client being motivated for participating in the intervention programme and being ready for making changes
2	If ABLE 2.0 is delivered in a context of goals defined by the referral service …	… it may activate limited involvement of the client …	… contributing to problems related to building a foundation for the entire intervention
	**Institutional level**		
3	If ABLE 2.0 is delivered in a context of supportive management …	… it may activate the OT feeling obliged and responsible …	… contributing to the OT feeling skilled and professional when delivering the programme
4	If ABLE 2.0 is delivered in a context of training and support, i.e., supervision and exchanging experiences among OTs	… it may activate the OT feeling confident in delivering the programme …	… leading to OT feeling satisfied and engaged
	**Interpersonal level**		
5	If ABLE 2.0 is delivered in a context where the client and the OT initially perceive to be ‘on wavelength’, share thoughts, and the client finds the OT to be professional …	… it may activate mutual confidence and openness …	… contributing to the OT being vigorous and client feeling his/her problems are being acknowledged
6	If a systematic approach is applied in the initial sessions, including a mandatory structured dialogue on eventual discrepancy …	… it may activate the client feeling confident and involved, perceiving to gain insight, and a collaborative relationship between the client and the OT …	… contributing to building a foundation for the intervention, initiating a problem-solving process, and client and OT gaining a common understanding of the client’s ADL ability
7	If ABLE 2.0 is delivered in the context of relatives being part of the client’s context of performance …	… it may activate the client being more/less engaged …	… contributing to a more/less facilitated process
	**Individual level**		
8	If ABLE 2.0 is delivered in the context of a skilled OT …	… it may activate a sense of believing in the programme and in the OT …	… contributing to the client finding content of programme meaningful
9	If ABLE 2.0 is delivered in the context of an OT feeling confident in explaining how and why the models are used …	… it may activate a fruitful communication and the client perceiving the OT to be professional …	… contributing to the client finding content of the programme meaningful and to establishing a foundation and agreement on focus for further intervention
10	If ABLE 2.0 is delivered in the context of a client with positive/negative expectations …	… it may activate the OT being more/less engaged …	… contributing to easier/constrained establishment of a therapeutic relationship

*ABLE* A Better Everyday Life (intervention programme), *ADL* Activities of daily living, *CMO* context mechanism outcome, *GAS* goal attainment scaling, *OT* occupational therapist

**Table 3:** Context-mechanism-outcome configurations (CMOCs): Establishing the focus for further intervention

CMOC no	Context	Mechanism	Outcome
	**Institutional level**		
11	If ABLE 2.0 is delivered in the home of the client ...	… it may activate an increased knowledge of the client’s everyday life and preferences, client feeling relaxed, flexible planning and timing, OT feeling obliged and responsible …	… contributing to client empowerment, defining relevant and occupation-focused goals, and future occupation-based intervention
	**Interpersonal level**		
12	If ABLE 2.0 is delivered in the context of collaboration and dialogue between client and OT, when clarifying causes for the ADL problems using a transactional perspective …	… it may activate involvement of the client in the analytic approach to understanding the ADL problems from a more transactional perspective (e.g., taking other perspectives than diagnosis into account) …	… contributing to more perspectives and new insights on the ADL problems, and to revealing new, other, and more ideas for solutions
	**Individual level**		
13	If ABLE 2.0 is delivered in the context of an OT being skilled in communicating and administrating GAS …	… it may activate a collaborative relationship, goal setting primarily based on the client’s priorities (including that the client has the power to define and formulate goals and levels of goal attainment) …	… contributing to establishment of basis for monitoring progress, and relevant and clear goals
14	If ABLE 2.0 is delivered in the context of an OT perceiving to lack skills administrating GAS …	… it may activate limited involvement of the client in defining goals and levels of goal attainment and in dialogue on causes for ADL problems …	… contributing to interruption of the logical order in the intervention, interruption of the coherence and the problem-solving process
15	If ABLE 2.0 is delivered in a context of a client with cognitive deficits …	… it may activate limited involvement of the client in dialogues related to goal setting, causes for ADL problems and in the overall problem-solving process	… contributing to less relevant and clear goals, lack of framing of the further intervention

*ABLE* A Better Everyday Life (intervention programme), *ADL* Activities of daily living, *CMO* context mechanism outcome *GAS* goal attainment scaling, *OT* occupational therapist

**Table 4:** Context-mechanism-outcome configurations (CMOCs): Identifying and implementing relevant compensatory solutions

CMOC no	Context	Mechanism	Outcome
	**Institutional level**		
16	If ABLE 2.0 is delivered in the context of the client’s home when finding solutions …	… it may activate the consultative process …	… contributing to finding effective and sustainable solutions
17	If ABLE 2.0 is delivered in the context of a system working on the client’s premises, including effective coordination between services and access to assistive devices …	… it may activate the client’s confidence with the system, and client and OT feeling successful …	… contributing to client feeling motivated for changes and feeling satisfied with the content of the sessions; and to solutions adjusted to the client’s context
18	If ABLE 2.0 is delivered in the context of the system’s terms …	… it may activate decreased confidence with the system’s ability to help and OT feeling powerless …	… contributing to interruption of the problem-solving process, decreased ADL ability, and decreased benefit of assistive devise
	**Interpersonal level**		
19	If ABLE 2.0 is delivered in the context of dialogue between client and OT; and the OT has a non-directive approach to this collaboration …	… it may activate the client feeling involved and having the power to accept or reject suggestions …	… contributing to client finding content of sessions meaningful
20	If ABLE 2.0 is delivered in the context of the OT observing the client trying out solutions during performance of ADL tasks …	… it may activate the OT acting as facilitator of change using an adaptational approach and the client feeling that his/her needs are legitimised …	…contributing to client feeling satisfied with content of sessions
	**Individual level**		
21	If ABLE 2.0 is delivered in a context of a client with insight in own ADL ability, and who can understand, remember, and maintain knowledge on his/her ADL ability and the causes for the ADL problems in focus (revealed during the first sessions) …	… it may activate the client being actively involved in the problem-solving process …	… contributing to finding relevant solutions and goal attainment
22	If ABLE 2.0 is delivered in the context of a client being open-minded to finding other solutions than he/she expected in advance …	… it may activate an occupation-based approach where the client is willing to try out solutions and the client and OT discuss and exchange ideas primarily based on the client’s priorities, and the client having the power to accept or reject solutions …	… contributing to client finding the programme content meaningful; motivation for staying in the programme; and finding focused, targeted, and potentially sustainable solutions
23	If ABLE 2.0 is delivered in the context of a client having applied for specific practical assistance, e.g., for cleaning …	… it may activate limited motivation for making other types of changes …	… contributing to challenges in finding relevant solutions
24	If ABLE 2.0 is delivered in the context of a client with cognitive deficits …	… it may activate limited involvement in the problem-solving process …	… contributing to limited benefit of intervention
25	If ABLE 2.0 is delivered in the context of an OT being empathetic, kind, skilled and competent …	… it may activate focused communication and collaboration between client and OT …	… contributing to finding relevant solutions and improved ADL ability

*ABLE* A Better Everyday Life (intervention programme), *ADL* Activities of daily living, *CMO* context mechanism outcome, *GAS* goal attainment scaling, *OT* occupational therapist

**Table 5:** Context-mechanism-outcome configurations (CMOCs): Re-evaluating ADL ability to finalise intervention

CMOC no	Context	Mechanism	Outcome
	**Institutional level**		
26	If ABLE 2.0 is delivered in the context of an OT conducting systematic re-evaluation using standardised instruments …	… it may activate clarity on obtained changes …	… contributing to demanded documentation
	**Interpersonal level**		
27	If ABLE 2.0 is delivered in the context of giving concrete feedback in terms of comparing level of obtained goals with level at session 1 …	… it may activate the client perceiving that the intervention made a difference and feeling motivated for integrating the new methods into everyday life routines …	… contributing to sustainable changes
	**Individual level**		
28	If ABLE 2.0 is delivered in the context of an OT being skilled in interpreting and explaining the results …	… it may activate the client getting insight in occurred changes and motivation for carrying on using the new strategies …	… contributing to sustainable changes

*ABLE* A Better Everyday Life (intervention programme), *ADL* Activities of daily living, *CMO* context mechanism outcome, *GAS* goal attainment scaling, *OT* occupational therapist

### Building a foundation for the entire intervention

Data reflected that during sessions 1 and 2 contextual factors at different levels facilitated or constrained the process of building a solid foundation for the entire intervention. Building this foundation was framed and structured by thorough evaluation of the client’s ADL ability, by actively involving the client in the process, and by taking the client’s perspective into account. CMOCs related to this theme are presented in Table 2.

At the infrastructural level (Table 2, CMOC no 1–2), the client’s pathway to rehabilitation service played a role in building a foundation for the entire intervention. As the IPT had no assumptions related to the impact of client’s pathway to rehabilitation, this will inform future refinement of the IPT. The following paragraph describes how.

In the municipality a client could be referred from the referral services (e.g., when applying for support in the home), or from the rehabilitation team (e.g., if a physiotherapist discovered that a client experienced ADL task performance problems). It was common practice in the municipality, that the referral service defined goals for the granted intervention. This tended to prevent building a foundation for the entire intervention, by counteracting the certain order of the content of ABLE 2.0, prescribing evaluation of ADL ability prior to goal setting. An OT said: “It is confusing for the clients, they expect us to work on [goals related to] bathing [as defined by the referral service], and then we also ask about dressing and cooking [as prescribed in the ADL-I] … the order of things in ABLE involves the client a lot more” (FG). The clients’ pathways affected motivation for participating in the intervention programme and readiness for making changes across ADL tasks. Hence, when a client was referred from the referral services, and goals were defined prior to initiating the occupational therapy intervention process and prior to evaluating the client’s ADL ability, building a foundation for the entire intervention was problematic.

At the institutional level (Table 2, CMOC no 3–4), the support from the management in prioritising time for training OTs in delivering ABLE 2.0 in accordance with the manual, and in legitimising deviations from usual practice (e.g., number or length of visits), facilitated the OTs feeling obliged and responsible. This, informing future refinement of the IPT, led to increased engagement in delivering what the OTs called ‘quality occupational therapy’, and to a sense of being skilled. Furthermore, the supportive management resulted in important support from colleagues in terms of accepting new ways of working, and in referring relevant clients to occupational therapy. Sometimes the OTs did however perceive lack of understanding of the new way of working among their colleagues, especially related to delivery of session 1, taking more time than a usual start-up. An OT said: “… of course the manager’s attitude [matters], the fact that you have an employer who thinks it’s important to deliver these interventions, and that we get enough time for it” (OT2). Another OT said: “Some of our colleagues said, well it was good you finished it [participating in the research] … they thought it took a lot of time and that we were less available …” (FG). Another contextual factor at the institutional level, confirming the IPT (Fig. 1a), was related to training and support in delivering the intervention, i.e., the three-and-a-half-day course, the exchange of experiences between the OTs, and the access to supervision on delivery from the research group, when challenges occurred. This activated the OTs feeling confident in delivering the programme, leading to OTs feeling satisfied and engaged. An OT said: “I have used her [the primary investigator] very much, to make sure I was on the right track. It has just meant a lot … I have also shared many things with my two colleagues involved in it [delivering ABLE] …” (OT1).

At the interpersonal level (Table 2, CMOC no 5–7), ABLE 2.0 provided a frame for building confidence and collaborative relationships between the client and the OT, overall confirming the IPT (Fig. 1a and b) in terms of triggering the therapeutic relationship as a mechanism. Such relationships were found core in building the foundation for the intervention process and led to satisfaction and engagement among both clients and OTs. A client said: “She was nice and straightforward, she listened to me, and I was straightforward too, and then we just got started … we were on wavelength right away, and that helped a lot” (C2). Administration of evaluations based on both self-report and observation of ADL ability at session 1 was found to be a prerequisite for initiating the problem-solving process. This systematic approach framing the first meeting between the client and the OT, activated involvement of the client. A client said: “I think it was really good, especially because of those schemes [AMPS and ADL-I] we used. … I even got an insight, thinking in a different way. We put it into words, whether I needed help, or it was hard or easy for me, whether I felt pain … I saw that yes, it is actually true that I need help” (C7). Data also showed, that when the OT was feeling skilled and engaged in delivering what they termed ‘quality occupational therapy’, e.g., using the instruments for evaluating the ADL ability, it led to the client feeling satisfied, engaged, listened to, seen, and understood. Further, this led to revealing the client’s perspective on his/her ADL ability. An OT said: “You feel well informed [after having conducted ADL-I and AMPS] to move forward, and you really feel you have established a common starting point to move forward, because we got in depth with the client’s everyday life …” (OT2). In addition, the ABLE 2.0 manual provided guidelines for identifying potential discrepancies between the client’s and the OT’s perspectives on the ADL ability. This dialogue was found to activate the client feeling confident in the collaborative relationship, leading to a common foundation for further intervention. This dialogue was especially important in cases where discrepancy occurred. An OT said: “Having both the client’s perspective and the therapeutic perspective, has a huge impact … it shows a very clear picture of the situation” (OT2). Further, data showing how relatives may have facilitated or constrained the intervention process will inform future refinement of the IPT. In one case, a spouse was ill and needed special care from the client, causing lack of energy to actively participate in ABLE 2.0, limiting the establishment of a foundation for the entire intervention. On the other hand, when a relative actively supported the process of a client by e.g., helping to describe how certain ADL problems occurred in the home, the intervention process was facilitated.

At the individual level (Table 2, CMOC no 8–10) the most influential contextual factors confirming the IPT (Fig. 1a and b) were the OTs being skilled and professional, activating a feeling among the OTs of delivering what they called ‘quality occupational therapy’, and a sense of believing in the impact of the programme. The skills that the OTs built during the three-and-a-half day course and the practising in delivering the programme, simultaneously improved their ability to communicate with the client about the different parts of the intervention, e.g., the instruments used for evaluation of ADL ability, and thereby actively involve the client. This will inform future refinement of the IPT (Fig. 1a). Hence, when the OT felt confident in explaining how and why the models or instruments were used, it activated a fruitful communication and the client perceiving that the OT was professional, leading to the client finding content meaningful, and to establishing a foundation and agreement on focus for further intervention. An OT said: “…being forced to professionally stick to the manual, to use those tools, and have to use some professional terms when communicating with the client …” (OT1). Furthermore, the client’s motivation and readiness for making changes, and his/her positive expectations, seemed to have activated mechanisms in terms of the OT being more engaged in the assessment of the ADL ability, leading to establishing therapeutic relationship as basis for further collaboration.

### Establishing the focus for further intervention

Data reflected that contextual factors at different levels facilitated or constrained the process of establishing the focus for further intervention, provided that the previously described foundation was built during the first sessions. A strength in ABLE 2.0 was perceived to be the coherence between the different parts, the logical order of the sessions and the way each step led to the next step. All together involving the client in the problem-solving process and establishing the focus for further intervention. The focus for the further process was primarily established during session 2, framed by using GAS for goal setting and PEO and/or TMO in clarifying causes for the ADL problems, including an active involvement of the client and taking the client’s perspectives into account. CMOCs related to this theme is presented in Table 3.

At the institutional level (Table 3, CMOC no 11), delivery of the intervention in the home of the client was important for establishing the focus for further intervention, promoting the OT’s knowledge of the client’s ADL ability, everyday life, and preferences; and affecting the client’s engagement and experience of meaningfulness. A client said: “She saw how I did things in my bedroom, in my own bed. That was good because I know how it works for me here” (C3). This confirmed the IPT (Fig. 1b and c), regarding the impact of delivering ABLE 2.0 in the client’s home. Delivery in the home of the client was considered the ideal context to facilitate a dialogue focusing on ADL task performance (i.e., occupation-focused dialogue), involving the client in an analytic approach, and in setting occupation-focused goals based on the client’s priorities. An OT said: “They were more relaxed in their own surroundings; it was the most natural setup, and it was always an advantage to be in the client’s home” (OT3). Discussing and determining the focus for the further process in the home of the client led to more knowledge on the ADL task performance problems and facilitated ideas for possible solutions. Hence, it pointed towards content in the future occupation-based (i.e., engaging the client in performing ADL tasks), intervention sessions. Further, delivering the interventions in the homes of the clients, supported the inherent element of flexibility in terms of how the OT planned and timed the intervention, facilitating the OTs feeling obliged and responsible, and the client being more relaxed, leading to client empowerment, fruitful dialogues, and relevant goals.

At the interpersonal level (Table 3, CMOC no 12), ABLE 2.0 provided a frame for focusing the further intervention by facilitating a collaborative and occupation-focused dialogue between the OT and the client, in defining goals and discussing causes for the ADL problems. This will inform future refinement of the IPT on the functioning of session 2 (Fig. 1c). In most cases the clients were actively involved in defining goals and levels of goal attainment, which activated the OT’s and the client’s reasoning, and served as a starting point for focusing the process. The OTs agreed: “You cannot conduct an ABLE intervention if you don’t use GAS or the other tools. It just would not work … you cannot get from A to Z if you do not use K or F. You must practice and practice and become proficient in using them” (FG). Further, they said: “GAS is a good tool. It is complicated to use though. And some clients are difficult to involve, especially those with cognitive deficits” (FG). When applied as intended, the goal setting process activated a dialogue on both parties’ notions of expected outcomes. This led to relevant and clear goals framing and targeting the intervention and establishing the basis for monitoring the progress. An OT said: “The levels [in GAS] helped me to think in steps and made it [the focus] clear to the clients. So, GAS helped to set the frame for the intervention and to align expectations” (OT2). Another OT said: “Most of my clients were really involved in defining the different levels … it became concrete … and at the end of the intervention it was easy to monitor” (OT3). In the context of discussing causes for the ADL problems, data showed that the use of models (i.e., PEO and TMO) offered an opportunity to move from a disease-oriented to a more transactional perspective on the client’s ADL problems. An OT said: “Many of the elderly tend to point to themselves [when talking about causes for ADL problems] saying, “It’s because I’m an old one”. Using the PEO model was a way of opening the dialogue on this. We could talk about other causes than those pointing at themselves” (OT3). Another OT said: “If you find it hard to explain to the client, then the model [PEO] helps you. Some clients never thought about other reasons than their disease. It becomes clear, how we can find resources in the environment, and they can find opportunities to be able to do the things they want to be able to do … this just means everything for the further focus” (OT2). Hence, the dialogue based on a transactional perspective led to involving the client in the problem-solving process. This was an eye opener for the client, and of great importance when establishing a focus for the intervention in terms of relevant and clear goals pointing towards potential compensatory solutions.

At the individual level (Table 3, CMOC no 13–15), client characteristics were influential, also pointing towards future refinement of the IPT. By applying GAS for goal setting, the OT was provided with a vocabulary to communicate with the client about setting goals. Hence, in the context of being a skilled OT mastering the use of GAS and involvement of the client, and having words to facilitate a dialogue on causes, the collaborative relationship between client and OT was activated, establishing the focus for further intervention. However, there were also cases, where involving the client in defining goals and levels of goal attainment failed (i.e., implementation failure). An OT said: “The main goal is fairly easy to define in collaboration with the client, but those sub-goals … it is something I usually do by myself, you know, the client says his or her main goal, and then I formulate the sub-goals, in relation to time, energy, risk of falling and those things [quality of performance]. I sometimes found it difficult to define in detail [the levels in GAS] with the client” (OT1). The OTs described that they sometimes perceived lack of skills in using GAS. This was amplified by the usual workflow in the municipality, implying that the OTs followed the goals defined by the referral service, and hence did not involve the clients in goal setting and/or in a dialogue on causes for ADL problems. When the implementation failure on goal setting occurred, there was a tendency that the intended problem-solving process was interrupted, as goals were formulated as concrete solutions (e.g., be able to vacuum the kitchen floor with a cordless vacuum cleaner) rather than as quality of performance (e.g., be able to vacuum the kitchen floor without risk of falling) as prescribed in the ABLE 2.0 manual.

The interviewed clients only rarely recalled the dialogue on goal setting. They recalled the focus for the intervention, but not the intended dialogue and formulation of levels in goal attainment. This might be due to examples of implementation failure in goal setting (e.g., the OTs sometimes did not include the clients in the goal setting process and formulated goals including the solution rather than the quality of performance to be attained).

### Identifying and implementing relevant compensatory solutions

Data revealed that contextual factors at different levels facilitated or constrained the process of identifying and implementing relevant compensatory solutions to enhance the client’s ADL ability, provided that the previously described foundation for the entire intervention was built and the focus for further intervention was established. Identification and implementation of relevant compensatory solutions was done during the intervention sessions (sessions 3–7). This was framed by the ABLE 2.0 intervention components and conducted in collaboration and dialogue between the client and the OT, actively involving the client in the problem-solving process, and by trying out possible solutions in the client’s home. CMOCs related to this theme is presented in Table 4.

At the institutional level (Table 4, CMOC no 16–18), the use of the environment (here the client’s home) facilitated the process of finding and trying out solutions, confirming the IPT (Fig. 1d). When the intervention sessions were delivered in the client’s home, it supported how the client could both explain and demonstrate issues related to his/her ADL task performance in the actual environment. Thus, the consultative process of finding effective and sustainable solutions was facilitated. Further, the Ots perceived that clients were less likely to cancel appointments, as they did not have to leave the home. An Ots said: “I think it [finding solutions in the home] gives them peace and makes them feel confident ... I do not find it possible to do it [practice solutions] in other ways … and when we come to them, there is a greater chance that they will accept it … if they have to come to us, we sometimes experience dropouts” (OT2). In addition, informing future refinement of the IPT, when the collaboration across the community-based organisation (i.e. rehabilitation service, referral service, assistive device service, home care service) was timed on the client’s premises and was experienced to be smooth and effective, the clients and the Ots felt that it was worth their effort, that solutions could be adapted to fit the client and client’s context, and that they were successful. This was satisfying and motivating for the client. For example, it was important to have access to a suggested assistive device. A client said: “It happened pretty fast. They came and lined them up [assistive devices]. I was completely surprised it happened so fast … I thought there was a wait for something like that. A lot of things happened … I am very happy about it” (C1). On the other hand, when ABLE 2.0 was carried out on the system’s premises, with delay in delivery of sessions due to a wait for assistive devices, it had consequences for the problem-solving process, for consultation of the client in using the assistive device, and for the client’s confidence with the system, potentially resulting in decreased benefit of the intervention. An OT said: “… the client may lose function and lose ability to use the assistive device or lose confidence in our help. Or, maybe they will need more home care.” (FG).

At the interpersonal level (Table 4, CMOC no 19–20), data revealed that collaboration, dialogue and discussion between client and OT were crucial and facilitated the process of finding and implementing solutions. Hence, several solutions were discussed and tried out to determine which to apply. Further, when the OT had a non-directive approach suggesting different solutions, it led to the client feeling actively involved in the problem-solving process and having the power to accept or reject suggested solutions and was associated with the experience that the content was meaningful. A client said: “We discussed it, whether it was the right solution” (C5). This will also inform refinement of the IPT (Fig. 1d). Several clients also highlighted the fact that the OT observed their ADL task performance during the problem-solving process, confirming the IPT (Fig. 1d). As the OT observed the client being engaged in e.g., watering flowers or cleaning the floor, she had the opportunity to suggest and guide in new ways of doing. One of the clients described this as an eye opener (C7). Another client expressed the value of being observed during engagement in ADL task performance like this: “I think it was good. Because as I said, talking does not do it alone. I prefer some action too.” (C1). When the OT observed the client’s performance, the clients sometimes considered it a validation of their needs which to some extend legitimised e.g., applications for assistive devices. One of the clients said: “I feel that there was really someone who could see that I needed it, that it was not just something I asked for.” (C7).

At the individual level (Table 4, CMOC no 21–25), the most influential contextual factors in identifying and implementing relevant compensatory solutions were related to the characteristics of the OTs and the clients. For example, that the OT was empathetic, kind, skilled and competent. This will inform future refinement of the IPT. The skills and competencies were primarily related to communication and collaboration on relevant solutions. One client said: “She was nice, kind, and straightforward, and we could just get to the point” (C5). Another client said: “She was nice and understanding, and she was on the marks when I complained about the toilet and the sheets, … I felt she heard me … and it was fixed right away” (C8). Further, based on the initial sessions in ABLE 2.0 the OTs had a solid foundation for planning and implementing interventions in a competent way. A client said: “It was the same scheme [ADL-I] we used every time, and then when she saw me do it [water my flowers], using my new chair, she could guide me. It was an eye opener … now I can just roll over to my flowers and fix it, and it does not hurt, when I do it anymore” (C7). Client characteristics in terms of motivation, readiness for making changes, and his/her expectations to the programme, were expressed to have an impact when finding solutions. A client said: “I was not expecting certain things [prior to the intervention], I was just waiting for what was going to happen … positive thinking you know … I am sure that meant a lot [for the benefit of the intervention]” (C5). And an OT said: “The clients’ motivation mattered to finding goals and solutions, to how I could help them make changes … and their engagement mattered a lot to the benefits” (OT2). These individual level contextual factors seemed to activate professionalism both experienced by the OTs and the clients, and a sense of joint commitment, informing future refinement of the IPT. Further, these factors lead to the OT being engaged in suggesting targeted and sustainable solutions adjusted to the specific client and his/her environment. Thus, potentially leading to improved ADL ability. On the other hand, when a client specifically had applied for help with, for example cleaning, the client’s motivation for finding other compensatory solutions, e.g., using assistive devices or changing the physical environment, was sometimes lacking, which was perceived to impede the collaboration on trying out different solutions. Further, when a client lacked insight, due to age or cognitive deficits, involving the client in the problem-solving process was a challenge. An OT said: “In a few clients, if they had decreased insight in their own situation … sometimes they had difficulties seeing the problems. Even though they had reported it in the ADL-I, still they did not remember it in the next sessions and when trying to find solutions” (OT3).

### Re-evaluating ADL ability to finalise intervention

The ABLE 2.0 IPT included assumptions concerning the functioning of the final session confirmed by data (Fig. 1a and e) and specifically data related to the instruments applied at the final session will inform future refinement of the IPT. Due to the study design, with evaluation of effectiveness conducted alongside this realist evaluation, the re-evaluation session was conducted somewhat different than originally intended in ABLE 2.0. Because AMPS was performed by blinded assessors as part of collecting primary outcome data for the RCT, the AMPS was optional at the final session, resulting in primarily performing re-evaluation based on the ADL-I and the GAS. Hence, data on the final session was limited. However, data reflected that contextual factors at different contextual levels facilitated or constrained the process of re-evaluation to finalise the intervention. CMOCs related to this theme is presented in Table 5.

At the institutional level (Table 5, CMOC no 26), ABLE 2.0 provided a frame for documenting changes in ADL ability, which informs future refinement of the IPT (Fig. 1e). The documentation based on the AMPS was especially useful when the clients applied for e.g., home care services and/or assistive devices. An OT expressed it this way: “The ADL-I … sometimes it can easily stand completely alone … and I can document without the AMPS. But it depends a lot on what the client is applying for … when I used AMPS [at the final session] it was because the referral service should make a decision on the client’s need for assistance in tasks related to cleaning …” (OT1). Further, one of the OTs expressed it like this: “Using the AMPS for re-evaluation is especially relevant when you need to document to the referral service or to the general practitioner or the nurse, and where I as OT can see, that even though we worked on this for eight weeks, nothing changed, and we need to apply for some assistance in the home” (FG). Moreover, the AMPS was found useful as documentation in the client records, in the case of future referral to rehabilitation services.

At the interpersonal level (Table 5, CMOC no 27), ABLE 2.0 provided a frame for re-evaluation of the client’s ADL ability by facilitating a dialogue between the client and the OT on goal attainment, obtained changes and ADL ability at the final session, confirming the IPT (Fig. 1e). Finalising ABLE 2.0, applying the prescribed instruments, had an impact on how to provide feedback to the client. The OTs agreed that GAS was the preferred instrument for providing feedback to the client on obtained changes, because it assessed the attainment of the specific goals in focus. In comparison, the ADL-I was found less relevant in terms of providing feedback, focusing on the ADL ability at the end of the intervention, but without comparison to the ADL ability at session 1. An OT said: “My experiences of using GAS [for monitoring attainment of goals] are good … it provided an awareness for the client on the current level and what was achieved” (OT2). Further, she said: “It was a bit harder for me to see the point in using it [ADL-I] in the final session … GAS is kind of a better summary for the client. In the ADL-I, I think, the clients are not asked about their experience of progress. We did not compare the scores [at the beginning of the intervention with scores and at the final session]. I also think the ADL-I was a little too comprehensive for the clients” (OT2). An OT explained how she experienced that ADL-I was less useful for providing feedback to clients: “Even though the intervention ran over several weeks, they still saw themselves as they functioned before the intervention. As if they had too little time to understand the implementation of their new habits” (OT3).

At the individual level (Table 5, CMOC no 28), ABLE 2.0 provided a frame for the OT to perform valid re-evaluation to finalise the intervention, confirming the IPT (Fig. 1a and e). When the final session was delivered in the context of an OT being skilled in interpreting the results, and when the OT supported the dissemination of the results to the client with visual material (e.g., the graph in the AMPS report), it activated the client’s insight in occurred changes and motivation for carrying on using the new solutions, potentially contributing to sustainable changes. An OT said: “The ADL-I is good, and in a few cases I also performed AMPS, using it to show them, how they did during these eight weeks. I prefer to use the graph from AMPS [from session 1], to compare with where they are now … it makes a huge difference” (OT1).

## Discussion

This realist evaluation aimed to explain in what circumstances, for whom, why and how ABLE 2.0 may or may not contribute to changes in ADL ability in persons living with chronic conditions. A total of 28 CMOCs were identified within four interrelated themes; building a foundation for the entire intervention, establishing the focus for further intervention, identifying and implementing relevant compensatory solutions, and re-evaluating ADL ability to finalise intervention. No aspects of the IPT were rejected, several were confirmed, and some aspects are to be refined. Overall, the study findings provide valuable information in further refinement of the ABLE 2.0 programme theory and in explaining the functioning of the programme. The in-depth knowledge about which contextual factors are necessary to activate the desired mechanisms will be beneficial in preparation for implementation of the ABLE intervention programme in community-based rehabilitation settings. Further, because ABLE 2.0 represents an occupational therapy intervention, based on a problem-solving process, the qualitative findings of the study expand our knowledge on how and in which circumstances occupational therapy interventions work.

### What works and how does it work?

Based on this realist evaluation, and supported by evidence [54, 55], it is recommended that ABLE 2.0 is delivered based on a systematic problem-solving process involving the client throughout the intervention, and including initial evaluation of the client’s ADL ability, followed by goal setting, clarification of causes for the ADL task performance problems, and identification of relevant solutions. The structure and content of ABLE 2.0 is composed of standardised instruments and conceptual practise models. In that respect, ABLE 2.0 does not differ from what can be implemented in any clinical occupational therapy practice and does not imply special knowledge or skills. However, ABLE 2.0 is unique in outlining how the underpinning occupational therapy theories, conceptual practice models and instruments are applied and how the content interdependently work together to provide a coherent client-centred individualised occupational therapy process. This is supported by the results of the ABLE 2.0 RCT, showing that ABLE 2.0, compared with usual occupational therapy, was effective in terms of obtaining sustainable changes in observed ADL motor ability at 26 weeks [44]. The present realist evaluation revealed that the mechanisms that were triggered were; active involvement of the client in the problem-solving process, a collaborative working relationship, mutual confidence between the OT and the client, and a consultative process applying an adaptational approach.

Emphasised by both clients and OTs the evaluation of ADL ability, using the AMPS and the ADL-I, was crucial in building a solid foundation for the entire intervention. This is in accordance with findings in previous studies related to the ABLE programme. In the previous feasibility study [26], the clients and OTs found the formal and standardised evaluations highly meaningful and supportive of client involvement in the process. Moreover, the previous pilot study [27] confirmed these findings with overall high scores on the impact of session 1 in clarifying focus for intervention and establishment of a good basis for further cooperation. Previous research also support the combined use of ADL evaluations based on both self-report and observation when evaluating the ADL ability among persons with chronic conditions [3, 4, 44, 56, 57]. More specifically, that self-report and observation provide distinct but related information about ADL ability as self-report represents the insider’s perspective (client perspective), and observation represents the outsider’s perspective (OT perspective). Further, this realist evaluation revealed that the mandatory dialogue on discrepancy was a core step in the coherent process in terms of both parties becoming aware of the other person’s perspective. Overall, the findings provide evidence to support the initial evaluation phase outlined in the OTIPM [45] and reflected in ABLE 2.0 session 1, including evaluation of ADL ability based on both self-report and observation, as basis for goal setting and intervention planning.

Applying GAS [42, 43] for goal setting and PEO [47] and/or TMO [45] when clarifying causes for the ADL problems was found core in establishing the focus for the further process. An implementation failure was however identified in relation to goal setting. Several explanations for the challenges related to goal setting may be considered. One explanation could be lack of experience among the OTs in collaborative goal setting, as goals typically were defined by the referral service in the municipality. Another reason could be lack of communication and collaboration skills among OTs, to involve the client in using GAS. Finally, the challenges could be related to lack of ability to involve clients with cognitive deficits in setting goals. The complexity related to goal setting among persons with chronic conditions is recognised [58–61]. Despite the challenges revealed in this realist evaluation, results also revealed the value of goal setting by using GAS, both related to establishing the focus for further intervention and re-evaluation. This is in agreement with Wade [62], claiming that goal setting is and should be a central feature in rehabilitation and should be a core competence of members of rehabilitation teams. Moreover, based on a systematic review by Vermunt et al. [58], it is specifically recommended to apply collaborative goal setting with elderly persons with chronic conditions. Further, the results revealed that the dialogue between the client and the OT on clarification of causes for their ADL task performance problems, by using the PEO [47] and/or the TMO [45] (during session 2) triggered a core mechanism of change and hence contributed to make ABLE 2.0 work. Using these models offered an opportunity to move from a disease-oriented to a more transactional perspective on the clients’ ADL task performance problems, facilitating the use of e.g., environmental opportunities or adaptive occupations to compensate for ineffective ADL task performance. The OTs found that focusing on the chronic conditions did not explain the client’s ADL task performance. As prescribed in the OTIPM [45], the OT needs to understand why the ADL task performance problems occur to help the client improve in ADL ability. In the transactional perspective on occupation, “occupation is a response to situational elements that naturally shape each other” [45]. Thus, by considering how situational elements affect the person’s ADL task performance, and by moving beyond understanding ADL task performance problems as solely individual problems, more efficient and potentially sustainable solutions can be identified. Hence, the transactional perspective [45] is suggested important in supporting the process of finding relevant and effective solutions. In other words, the use of PEO and TMO supported focussing on ADL task performance during goal setting and intervention, which is in contrast to the biomedical model characterized by goals related to absence of disease and/or symptoms [63].

During the ABLE 2.0 intervention sessions, compensatory solutions were implemented. Due to ineffective ADL task performance, this means that the client may need to perform the task in a way that is different from what is usually considered typical. Thus, the OT often had the client “try out, practice, and learn to use their chosen adaptational strategies and ensure that they will be able to incorporate them into their daily life routines” [45]. Hence, compensatory solutions involve habit changes, and when aiming for sustainable changes even habit formation. In the previous feasibility and pilot studies [26, 27], ‘changing habits’ was a frequently implemented intervention component. Modifying habits by making changes in the physical or social contexts has previously been suggested to be the most effective and straightforward way of disrupting, developing, or changing habits [64, 65]. In ABLE 2.0 this is extended to also include adapting the task e.g., simplifying the task. This may lead to more efficient task performance in terms of reducing physical effort, contributing to finding potentially sustainable solutions. In a study conducted among women with diabetes, Fritz [66] found that implementing such habit changes required facilitating clients’ understanding of what they already do, rather than telling them what to do differently. Fritz also found that behavioural changes were initiated in inquiry but integrated through practice. This is also reflected in the problem-solving process of ABLE 2.0, underpinning the importance of the logical order of content (i.e. assessment prior to goal setting and dialogue on causes) and the consultative and educational process (i.e. engaging the client in decision-making and finding strategies on how to use the chosen compensatory solutions) [45]. Further, the fact that persons vary in their capacity to make contextual changes themselves [66] and that many people need assistance identifying deficits and potential solutions [64, 65] adds to the complexity in interventions aiming to enhance the ADL ability among persons with chronic conditions.

Overall, ABLE 2.0 was perceived to contribute to establishing therapeutic relationships and empowerment of the clients. Still, the present study also revealed that the OTs across sessions sometimes were challenged in communicating and collaborating with clients, suggesting a need for a variety of different therapeutic skills during delivery of the programme. Delivering ABLE 2.0 is not simply applying the tools, instruments, and models prescribed in the ABLE 2.0 manual [67]. The impact is found in the way OTs deliver the ABLE 2.0. Recognising the challenges in goal setting, future research activities related to the ABLE intervention programme should address the OTs’ skills in communicating around goal setting, and how to intentionally develop a fruitful therapeutic relationship building on mutual confidence during delivery of ABLE 2.0. The OTIPM [45] emphasizes that the collaborative working relationship between the client and the OT is a critical component of the therapeutic process. This is in line with the Intentional Relationship Model (IRM) describing six distinct ways, i.e. therapeutic modes (i.e. advocating, collaborating, emphasising, encouraging, instructing, and problem-solving mode) of relating [68]. For example by utilising the advocating mode, reflecting that the OT speaks for the client’s rights and help to secure resources [68] may be appropriate in case of inappropriate wait for assistive devises or home care; or when involving the client in finding relevant solutions the collaborating mode, reflecting that the OT works on an egalitarian level with the client, entrusting that the client lead the decision-making process [68] may be particularly appropriate. Hence, the IRM may be useful in supporting establishment and obtaining full benefit of the collaborative working relationship.

### For whom does it work?

The study indicated that ABLE 2.0 primarily worked for clients with positive expectations, who were open-minded towards, and perceived to be ready for, making changes. Clients, who had applied for specific assistance, e.g., assistance with cleaning, and in cases where goals were set by the referral service, were perceived to be less open-minded for implementing other solutions in relation to ADL task performance. Further, it was revealed that clients with cognitive deficits were less able to be involved in the problem-solving process and in finding relevant solutions. Finally, it was found that clients who could maintain what was found and discussed during the initial sessions were more likely to benefit. Recognising the challenge of proper involvement of some clients in a collaborative problem-solving process, this study stresses the importance of OTs possessing effective collaborative and communication skills when delivering the ABLE 2.0, especially, when collaborating and communicating about goal setting and clarification of causes for the ADL problems. Based on a conceptual review of engagement in healthcare and rehabilitation Bright et al. [69] found, that client engagement is a multi-dimensional construct, comprising both a co-constructed process and a client state, suggesting that while engagement is commonly considered a patient behaviour, clinicians play a pivotal role in client engagement. Our findings, supported by the findings of Bright et al. [69], suggest that the OT play an important role engaging clients from the onset of the ABLE 2.0, and to see the client engagement as something that is constructed in the therapeutic relationship and during the intervention programme.

### In what circumstances does it work?

Several contextual factors enabled that ABLE 2.0 provided a frame for enhancing the ADL ability among the participating clients, and hence are suggested to be prerequisites for successful implementation of the ABLE intervention programme. Contextual factors, including referral procedures encouraging the coherent problem-solving process, supportive management, a system working on the client’s premises, delivery in the client’s home, and skilled OTs triggered the identified mechanisms of change.

Reflected in the implementation failure on goal setting it was clear, that if one part was left out of ABLE 2.0, e.g., not involving the client in defining goals at session 2, the problem-solving process was problematic. Thus, the assumptions on the impact of the systematic approach in ABLE 2.0 was overall confirmed and it stands out that contextual factors supporting coherence between the different parts of the programme, and the logic order of the sessions in ABLE 2.0, were of particular importance. This is in line with the results of the ABLE 2.0 RCT [44], suggesting that the systematic approach by means of the OTIPM [45] seemed to be beneficial in enhancing ADL ability in people with chronic conditions. Hence, ABLE 2.0 should be delivered by trained, skilled, and engaged OTs, being capable of explaining the purpose, using the prescribed tools, and actively involve clients in the problem-solving process. The previous feasibility study [26] as well as the pilot study [27] revealed that the OTs perceived to be highly confident in delivering the initial sessions, being core in building a foundation for the entire intervention. Hence, the priority of establishing these skills during the three-and-a-half-day training course is suggested important in case of future implementation in other contexts. Further, both the ABLE 2.0 manual, access to supervision, and discussion with colleagues also seemed to be beneficial supporting the OTs in being vigorous, responsible, and confident during delivery. Second, the referral procedures played a central role in encouraging the coherent problem-solving process. Knowledge on how referral procedures themselves can be a barrier for delivering an intended rehabilitation programme seems limited. In a systematic scoping review on barriers and enablers to rehabilitation referral within chronic obstructive pulmonary disease Milner et al. [70] found that a common barrier was low knowledge of the benefits provided by the rehabilitation programme. Providing more knowledge on ABLE 2.0, in case of future implementation, is therefore suggested to overcome potential challenges related to the relations between referral procedures and delivery of ABLE 2.0. Further, future implementation of ABLE 2.0 may include adaptation of the local referral procedures to facilitate referral of eligible clients without affecting the problem-solving process negatively. It is also recommended to investigate these issues and their impact on the coherent problems-solving process as part of future implementation studies. Third, supportive management in the municipality ensuring resources demanded for delivering this new programme and ensuring acceptance among colleagues regarding its implementation in the study period, was found important for the OTs’ commitment and responsibility in delivering ABLE 2.0. Caldwell et al. [71] found that managers’ actions can facilitate implementation and reduce barriers to change, e.g., by communicating clearly and directly, by ensuring the needed knowledge and resources, by serving as facilitators, and by building a culture among staff where quality improvement is an expectation. Hence, managers play a key role in future implementation of ABLE 2.0 in existing rehabilitation settings.

Delivery of ABLE 2.0 in the client’s home was a consistent contextual factor, reported by clients and OTs to be the ideal setting contributing to increase the OT’s knowledge of the client’s everyday life and preferences, the client feeling more relaxed, and flexible planning and timing of the process. The home as setting allows to practice and implement new compensatory solutions immediately, which is in line with Hand et al. [54] suggesting individualised programmes and efforts for persons with chronic conditions to promote continued use of new strategies, e.g., by practising performance. Further, this confirms the relevance of the transactional perspective on occupation [45, 46] permeating ABLE 2.0, that occupation (here ADL tasks) is considered a response to several situational elements, including environmental, sociocultural, task, and temporal elements. It therefore matters, that engaging the clients in occupational performance during evaluation of ADL ability, and in practising the chosen compensatory solutions, takes place in their own surroundings.

## Strengths and limitations

Several limitations are to be considered. The study was conducted in a single centre, limiting the evidence of how the ABLE 2.0 functions in various contexts. Another limitation occurred due to the parallel conduction of an RCT, preventing interviews to be conducted immediately after delivery of sessions. Instead, interview data were collected several weeks after the inclusion of the client. Hence, the interviewed clients were generally challenged recalling details on the content of their interventions and on mechanisms, specifically concerning goal setting and clarification of causes for ADL task performance problems.

The client sample was to represent the heterogeneous target group of ABLE 2.0. Representativeness was achieved in terms of diagnoses, sex, age, and level of ADL motor ability at baseline. However, in terms of variation in outcomes the included clients overall reached the expected level of goals, which may represent a problem in gaining nuanced information on client experiences of whether ABLE 2.0 encouraged them to make changes in reasoning and/or behaviour in relation to ADL task performance. However, the clients delivered valuable information on how they perceived the focus of their intervention, how they felt about the OT, and how they perceived the process and the solutions applied during the intervention. In future studies it would be relevant to conduct client interviews immediately after a session, or alternatively to conduct focus group interviews with a client group selected for gaining information on mechanisms of change.

It was considered a strength that both clients having received, and OTs having delivered ABLE 2.0 was interviewed. Further, the use of programme theory, the longitudinal design, and the application of realist principles in terms of the teacher-learner function applied in the interviews, strengthened the study in providing valuable information on the functioning of the ABLE 2.0. The previous studies conducted within the ‘A Better Everyday Life’ research programme [23, 26], informed the development of the IPT, serving as structure for data collection and analysis. The IPT included very limited assumptions regarding infrastructural and institutional level contextual factors of impact, limiting the opportunity to investigate these contextual factors. However, the present study revealed comprehensive information on the impact of the infrastructural and institutional level contextual factors, considered to be of great importance in case of future implementation. Based on the IPT, expressing the ideas of how the intervention was assumed to work, qualitative interviews were conducted with persons receiving and delivering the intervention. The results based on the qualitative interview data were then compared with existing evidence. This reflects triangulation, resulting in comprehensive knowledge about the functioning of the ABLE intervention programme. Conclusively the methods applied in this study were helpful in revealing knowledge about how occupational therapy, delivered as ABLE 2.0, should be delivered and received, to obtain changes in occupational performance among people with chronic conditions.

## Conclusion

This study investigated in what circumstances, for whom, how and why the ABLE 2.0 intervention programme functioned in a Danish community-based rehabilitation setting. The ABLE 2.0 IPT was overall confirmed. Based on the study it is concluded, that when ABLE 2.0 is delivered within supportive municipal frames by skilled and engaged OTs in the home of a client feeling ready for making changes, and when compensatory solutions to resolve the ADL task performance problems are applied, a collaborative working relationship between the client and the OT could be established and sustainable changes in the clients ADL ability seem obtainable. With that respect, ABLE 2.0 represents a coherent problem-solving occupational therapy process, applicable across sex, age, and diagnoses, that has the potential to enhance the ADL ability among persons with chronic conditions, when delivered as part of community-based rehabilitation services.

The results of this realist evaluation provide valuable and nuanced explanations on how and in which circumstances the ABLE 2.0 may improve the ADL ability among people with chronic conditions. This in-depth knowledge about which contextual factors are necessary to activate the desired mechanisms in the ABLE 2.0 adds to the existing knowledge related to the functioning of ABLE 2.0 and will be beneficial in case of future implementation of the programme in routine practice in community-based rehabilitation settings.

Finally, using the principles of realist evaluation, the study contributed to the understanding of how occupational therapy, delivered as a coherent problem-solving process may improve performance of ADL tasks. Hence, it serves as an example of how to use the principles of realist evaluation to investigate the functioning of a complex intervention.

## Data Availability

The datasets used and/or analysed during the current study are available from the corresponding author on reasonable request.

## Abbreviations

ABLE: A better everyday life
ADL: Activities of daily living
ADL-I: Activities of daily living interview
AMPS: Assessment of motor and process skills
C: Client
CMOC: Context-mechanism-outcome configuration
FG: Focus group interview
GAS: Goal attainment scaling
IPT: Initial programme theory
IRM: Intentional relationship model
OT: Occupational therapist
OTIPM: Occupational therapy intervention process model
PEO: Person-environment-occupation
RCT: Randomised controlled trial
TMO: Transactional model of occupation
